# Analysis of Stop Codons within Prokaryotic Protein-Coding Genes Suggests Frequent Readthrough Events

**DOI:** 10.3390/ijms22041876

**Published:** 2021-02-14

**Authors:** Frida Belinky, Ishan Ganguly, Eugenia Poliakov, Vyacheslav Yurchenko, Igor B. Rogozin

**Affiliations:** 1 National Center for Biotechnology Information, National Library of Medicine, National Institutes of Health, Bethesda, MD 20894, USA; frida.belinky@nih.gov (F.B.); ganguly1708@gmail.com (I.G.); 2 National Eye Institute, National Institutes of Health, Bethesda, MD 20892, USA; poliakove@nei.nih.gov; 3 Life Science Research Centre, Faculty of Science, University of Ostrava, 710 00 Ostrava, Czech Republic; 4 Martsinovsky Institute of Medical Parasitology, Tropical and Vector Borne Diseases, Sechenov University, 119435 Moscow, Russia

**Keywords:** in-fame stop codon, expression, short-term evolution, population polymorphism, negative selection

## Abstract

Nonsense mutations turn a coding (sense) codon into an in-frame stop codon that is assumed to result in a truncated protein product. Thus, nonsense substitutions are the hallmark of pseudogenes and are used to identify them. Here we show that in-frame stop codons within bacterial protein-coding genes are widespread. Their evolutionary conservation suggests that many of them are not pseudogenes, since they maintain dN/dS values (ratios of substitution rates at non-synonymous and synonymous sites) significantly lower than 1 (this is a signature of purifying selection in protein-coding regions). We also found that double substitutions in codons—where an intermediate step is a nonsense substitution—show a higher rate of evolution compared to null models, indicating that a stop codon was introduced and then changed back to sense via positive selection. This further supports the notion that nonsense substitutions in bacteria are relatively common and do not necessarily cause pseudogenization. In-frame stop codons may be an important mechanism of regulation: Such codons are likely to cause a substantial decrease of protein expression levels.

## 1. Introduction

Most single nucleotide substitutions in protein-coding genes either change an encoded amino acid or are synonymous. These two types of substitutions are frequently used in measures of molecular evolution [1]. Another type of substitutions, nonsense mutations, is an understudied phenomenon. Nonsense mutations, by definition, turn a coding (sense) codon into a stop codon that is assumed to result in a truncated protein product. Thus, in-frame stop codons are the hallmark of pseudogenes and are used to identify them [2,3,4,5]. Nonsense mutations are potentially highly deleterious, and functional protein-coding genes are not expected to have them at all. Nonetheless, pseudogenes (frequently defined as protein-coding genes with in-frame stop codons) in pro- and eukaryotic genomes persist on the evolutionary timescale, implying that they are maintained by natural selection [6]. In addition, pseudogenes can be transcribed and translated [3,7].

The translation of pseudogenes is not a paradox that molecular biology cannot explain. For example, naturally isolated *Escherichia coli* strains display a wide range of ribosomal fidelity, suggesting that a high rate of translational errors may be favored under some conditions [8]. It was suggested that increased translational errors (including readthrough events) paradoxically provide benefits to microorganisms experiencing environmental stress [9,10,11,12]. For example, amino acid misincorporation in the β subunit of RNA polymerase increases resistance of mycobacteria to rifampicin [13,14], and translational errors increase bacterial tolerance to oxidative stress by activating their general stress response [15,16]. Interestingly, in such cases, only subpopulations of genetically identical cells survive severe stresses [14,15], suggesting that stress response activated by translational errors may be heterogeneous (noisy) at the level of individual cells. However, the origin and extent of such heterogeneity remain unknown. A recent study has suggested that fluctuations in the concentrations of translational components lead to UGA readthrough heterogeneity among single cells, which enhances phenotypic diversity of the genetically identical population and facilitates its adaptation to changing environments [17].

In addition, it is well documented that certain stop codons in species across all domains of life are reassigned to sense codons [18,19,20]. In such cases, it is obvious that a mutation toward a reassigned stop codon is not considered a nonsense mutation and, therefore, has a much-reduced effect on fitness. Interestingly, it has been suggested that there are defined evolutionary steps leading to stop reassignment, including a stop codon that becomes non-essential, as is the case for UAG in *E. coli* [21]. The non-essentiality of UAG does not mean that it does not function as a stop codon, but rather that genes that end with UAG are either less important or have downstream stop codons that can compensate for readthrough events. As a part of the evolutionary reassignment process, it is imperative to have a tRNA recognizing a stop as a sense codon [18,21]. Historically, such tRNAs have been identified as suppressor tRNAs, allowing for a readthrough of a stop codon [22]. It is also possible that regular tRNAs with lack of perfect codon–anticodon interactions would still have some affinity to stop codons and facilitate some level of readthrough events [23]. Recent advancements have provided a solid basis for the development of various experimental tools that are based on the incorporation of biologically occurring or chemically synthesized non-canonical amino acids into the recombinant proteins and even proteomes via reprogrammed protein translation [24]. This takes place in the frame of a greatly expanded genetic code with a variety of codons liberated from their current specificities [24]. An example of such expansions has been documented in some methanogenic archaeal species that synthesize proteins containing selenocysteine or pyrrolysine encoded by stop codons [25].

Here we show that stop codons within protein-coding genes are widespread in bacteria and are found in same positions of orthologous genes. This evidence of evolutionary conservation indicates that many of them are not pseudogenes, because they maintain dN/dS values downstream, to the stop codon significantly lower than 1. We also found that double substitutions, where an intermediate step is a nonsense mutation, show accelerated rates of evolution, as compared to null models, indicating that a stop codon was introduced and then changed back to a sense codon via positive selection. This further supports the notion that nonsense substitutions in bacteria are relatively common and do not necessarily cause pseudogenization.

## 2. Results

### 2.1. Large-Scale Study: Analysis of Complete Bacterial Genomes

The initial database searches revealed many potential stop codons in protein-coding genes (Supplementary Figure S1); however, we expect that a substantial fraction of them may have resulted from various artifacts (Supplementary Figure S2).

**Figure 1:**
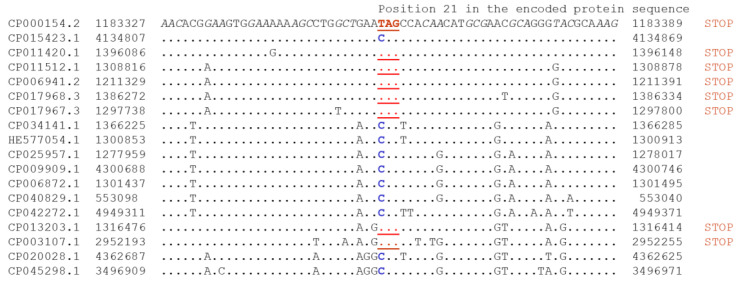
Partial alignment of the genes encoding the conserved 3′-phosphoadenosine 5′-phosphosulfate sulfotransferase protein family of *Paenibacillus* spp. The encoded protein sequence is conserved in over 100 *Paenibacillus* spp. (we used BLASTP search in non-redundant protein NR database with default parameters, https://blast.ncbi.nlm.nih.gov/Blast.cgi (accessed on 11 February 2021)). Analyses of surrounding genes at NCBI Genomes website (https://www.ncbi.nlm.nih.gov/genome/ (accessed on 11 February 2021), three genes upstream and three genes downstream) did not reveal any obvious conserved gene neighborhood.

**Figure 2:**
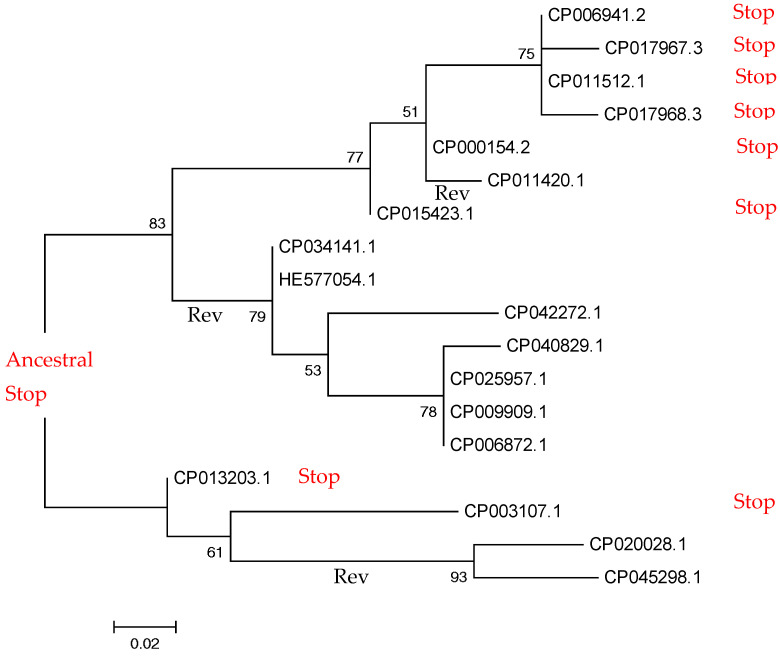
Molecular phylogenetic analysis by maximum-likelihood method. The tree with the highest log likelihood (−247.8) is shown. “Stop” indicated TAG codons, unmarked terminal branches contain “CAG” in the orthologous positions (Figure 1). “Rev” under corresponding branches indicates TAG > CAG substitutions. The bootstrap support values are shown next to the branches; the scale bar indicates the number of substitutions per site. The list of genomes is shown in Supplementary Table S1. The nearly perfect correspondence of the reconstructed (the current figure) tree and the species tree (Figure 2 in [26]) is a well-known property of the vertically inherited sequences [27] and suggests the absence of horizontal gene transfer events in the set of studied sequences (Figure 1).

To minimize those artifacts, we focused on stop codons that are present within orthologous protein-coding genes shared by two or more bacterial species (exemplified in Figure 1, for a conserved 3′-phosphoadenosine 5′-phosphosulfate sulfotransferase protein family of *Paenibacillus* spp.; Supplementary Figure S3). In-frame TAG stop codons are in the same position of the alignment (orthologous stop codons) (Figure 1). We use this example to illustrate problems associated with the analysis of in-frame stop codons. There are eight instances of TAG stop codon and ten instances of CAG (Figure 1 and Figure 2).

To delineate evolutionary history of the in-frame stop codon, we mapped TAG/CAG on the maximum-likelihood phylogenetic tree, which was reconstructed using nucleotide sequences (Figure 2). The most parsimonious scenario involves three TAG>CAG reversals, assuming that stop codon was ancestral (if CAG was present in the last common ancestor, two CAG>TAG and two TAG>CAG changes would be needed). In both scenarios the impact of reversal is substantial. It should be noted that the analyzed region is likely to be under purifying selection, as expected for protein-coding genes (mean dN = 0.124, mean dS = 0.170, where dN is the number of nonsynonymous substitutions per nonsynonymous site and dS is the number of synonymous substitutions per synonymous site). The dN/dS value below 1 (0.73 in this case) is an indicator of negative selection.

**Figure 3:**
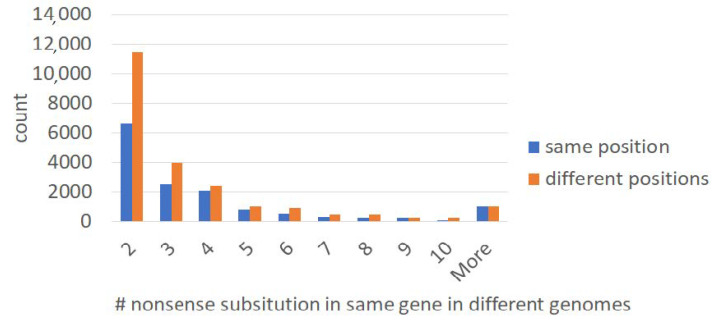
The number of orthologous and “non-orthologous” stop codons (located in different positions of the same protein family) in two or more genomes.

We estimated the fraction of independent occurrences of orthologous in-frame stops by comparing the number of orthologous and “non-orthologous” (those located in different positions of the same protein family) stop codons (Figure 3). The average fraction of codons (in all protein-coding genes used in this study) that can produce a stop codon as the result of single substitution (stop-codon-prone) is 22%. We used 600 codons (200 amino acids) as a lower bound estimate of a protein-coding gene’s length [28]. Moreover, 22% of 600 codons (132 codons) are stop-codon-prone. We observed a twofold excess of “non-orthologous” stop codons (Figure 3). Thus, the fraction of independent events among all “orthologous” stop codons is roughly 2/132 = 0.015. It should be noted that this is a conservative estimate. This indicates that the fraction of independent events (polyphyletic stop codons) is small and the vast majority of orthologous in-frame stop codon substitutions occurred just once.

**Figure 4:**
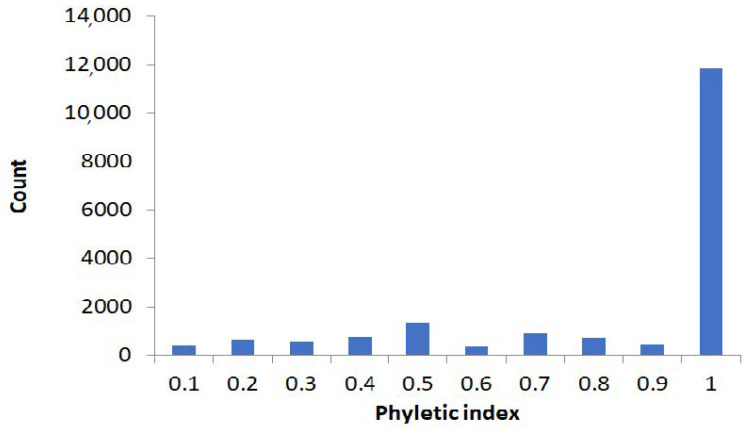
Distribution of the phyletic indexes of nonsense substitution on species trees.

We decided to define polyphyletic and monophyletic stop codons, using a conservative “phyletic index” approach illustrated in the Supplementary Figure S4: If two stop codons were separated by branches that have codons other than stop in the same position of an alignment, we assumed that they evolved independently. The distribution of the phyletic indexes is shown in the Figure 4. A fraction of polyphyletic stop codons is small, confirming that independent origin of “orthologous” in-frame stop codons is, indeed, unlikely.

Analysis of the number of mismatches around in-frame stop codons (window of 30 bases) suggested that the vast majority of pairwise comparisons have either a small number or no mismatches (71% pairwise alignments without mismatches, 15% with one mismatch, 7% with two mismatches, 3% with three mismatches, and 4% with more than three mismatches). This result strongly suggests that in-frame stop codons tend to persist for short evolutionary time at the scale of population polymorphism.

Protein-coding genes are expected to be under purifying selection (dN/dS < 1) (the ratio of the number of nonsynonymous substitutions per non-synonymous site to the number of synonymous substitutions per synonymous site, which can be used as an indicator of selective pressure acting on a protein-coding gene) [1,29,30]. If sequences with stop codons are pseudogenes or represent errors of annotation, no purifying selection is expected. The dN and dS values are shown in the Table 1. All three types of in-frame stop codons appeared to be under purifying selection (*p* < 0.001). Nevertheless, the dN/dS values are fairly high, suggesting that either a fraction of sequences are true pseudogenes or in-frame codons represent single nucleotide polymorphisms (SNPs): dN/dS values for negatively selected genes are typically closer to 1 when comparing intra-specific samples, as opposed to inter-specific samples [29].

**Figure 5:**
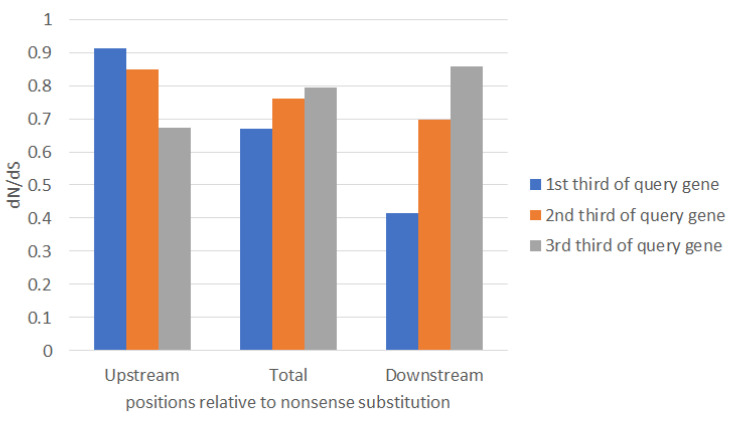
Graph of dN/dS values before and after stop codons in the first, second, and third terciles of studied genes with in-frame stop codons. Deviations from 1 were significant for all dN/dS values (*p* < 0.001).

Next, we analyzed dN/dS values before and after stop codons in the first, second, and third terciles of studied genes with in-frame stop codons (Figure 5). The high dN/dS in regions before in-frame stop codons in the first tercile may reflect problems with 5′ end annotations (Supplementary Figure S2), whereas high dN/dS after in-frame stop codons in the third tercile may reflect variability of 3′ ends [30].

Analysis of ATGC-COG functional categories did not reveal any major trends, except for an over-representation of the [X} “Mobilome: phages and transposons” (Supplementary Table S2) (9889 cases in total). It should be noted that the total number of cases of the next two most abundant categories ([G] “Carbohydrate transport and metabolism” [6126 cases] and [R] “General function prediction only” [5516 cases]) is substantially greater than the [X] functional category (12,713 vs. 9889) (Supplementary Table S2).

### 2.2. Small-Scale Study: Analysis of Stop Codons in Triplets of Species

We also performed a small-scale study in well-defined conditions that we used earlier, to study various types of substations [31,32,33]. It should be noted that signatures of positive selection have been found for double substitutions in stop codons in bacteria (UAG > UGA and UGA > UAG), which could be attributed to the deleterious, non-stop intermediate state, UGG [34]. We applied a similar model for analysis of in-frame stop codons; however, they are considered as intermediate steps in the current study.

**Figure 6:**
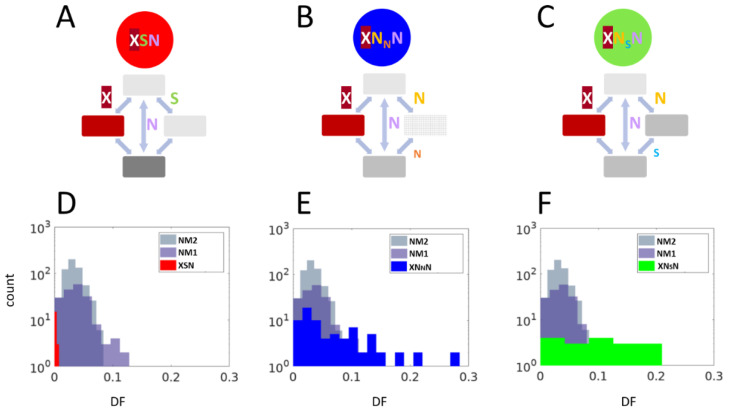
Types and distribution of double fraction (DF) relative to controls. (**A**) Double-substitutions XSN, in which a single substitution can be either synonymous or nonsense, while the double substitution is nonsynonymous, as compared to the ancestral state. (**B**) Double-substitution XN_N_N, in which a single substitution can be either nonsynonymous or nonsense, while the double substitution is nonsynonymous to the ancestral state, and the substitution between the intermediate nonsynonymous to the final nonsynonymous is nonsynonymous, as well. (**C**) Double-substitution XN_S_N, in which a single substitution can be either nonsynonymous or nonsense, while the double substitution is nonsynonymous to the ancestral state; however, the intermediate state is synonymous to the final state. (**D**) Distribution of DF values of XSN double substitution, compared to NM1 (syn_31) and NM2 (syn_33) control null models. (**E**) Distribution of DF values of XN_N_N double substitution, compared to NM1 and NM2 control null models. (**F**) Distribution of DF values of XN_S_N double substitution, compared to NM1 and NM2 control null models.

Using triplets of genomes with reliable phylogenetic relationships, we calculated frequencies of double and single substitutions in codons, and in double synonymous controls (Supplementary Figure S5). An important distinction of this approach is in use of double synonymous substitutions that served as null models for the double substitutions in codons. This control is important because some replication/repair enzymes are known to produce excessive numbers of simultaneous double substitutions [35,36,37]. Therefore, we compared the frequencies of all codon double substitutions to all possible types of double synonymous substitutions that were captured in two null models (Supplementary Figure S6). The first null model (NM1, syn_31) included a synonymous substitution in the third position of a codon, followed by another synonymous substitution in the first position of the next codon. The second null model (NM2, syn_33) included non-adjacent synonymous substitutions in third codon positions of consecutive codons. We found that the double fraction (DF), i.e., the observed double substitution frequency divided by sum of the cumulative single substitution frequency and the double frequency (Supplementary Figure S5), was typically higher for the syn_31 model, compared to that of syn_33 model, suggesting a mutational bias toward double substitutions in adjacent positions (Figure 6). The DF is assumed to be proportional to the second-step substitution rate (Supplementary Figure S6). If the elevated DF of codon double substitutions results solely from a multi-nucleotide mutational bias, the comparison to the null model is expected to show no significant difference. Conversely, a significantly lower DF compared to that of the null model is indicative of purifying selection, whereas a significantly higher DF points to positive selection. For example, the double substitution CAG > TCG can be the result of changes two consecutive substitutions CAG > CCG > TCG. Another pathway CAG > TAG > TCG contains the stop codon TAG and was not included in calculations. There are 201 double-substitutions CAG > TCG and 1288 single-substitutions CAG > CCG. The numbers of corresponding synonymous substitutions (NM1, syn_31 model) are 39 and 1023; thus, the excess of double substitutions associated with in-frame stop codons is significant (Supplementary Table S3).

Representing all within-codon double substitutions as “ancestral-intermediate-final”, we define the following three combinations, without taking into account the “stop codon intermediate” path (Figure 6A–C): (i) a double-substitution XSN, in which the first single substitution is synonymous, while the double substitution is nonsynonymous, compared to the ancestral state; (ii) a double substitution XN_N_N, in which the first single substitution is nonsynonymous, while the double substitution is nonsynonymous to the ancestral state, and the substitution between the intermediate nonsynonymous to the final nonsynonymous is nonsynonymous, as well; (iii) a double substitution XN_S_N, in which the first single substitution is nonsynonymous, while the double substitution is nonsynonymous to the ancestral state, however the intermediate state is synonymous to the final state.

The XSN changes are subject to purifying selection (Figure 6D and Supplementary Table S3), whereas many XN_N_N and XN_S_N changes are likely to have accelerated rates of evolution compared to neutral controls (Figure 6E,F). These results suggest that at least some double mutations have accelerated rates of evolution, due to the escape from in-frame stop-codon state. It should be noted that the analyzed triplets of species have the divergence rate of over 5%, and predicted mutations are unlikely to represent population polymorphisms (fixed mutations) [29]. The overall number of events is not high: For XNnN, we detected 12 positively selected versus 2 negatively selected cases; for XNsN, we detected three positively selected and no negatively selected cases (Supplementary Table S3). This is likely to be the result of small sample sizes.

## 3. Discussion

In general, the results of small- and large-scale analyses are consistent. According to the large-scale experiment, a vast majority of detected orthologous stop codons tend to exist for a short period of time (roughly corresponding to SNPs). Analyses of dN/dS (Table 1) are consistent with this time estimate. The accelerated evolution of some double mutations with an intermediate in-frame stop codon (Figure 6) reflects potential avoidance of in-frame stop codons for prolonged period of times. Indeed, the small-scale analysis was designed to study fixed mutations at the level of different species rather than SNPs [31,32,33]. Overall, our results suggest that in-frame stop codons and readthrough events (suppression of in-frame stop codons) are likely to be widespread biological phenomena in prokaryotes, and such stop codons could be advantageous for short periods of time.

We were able to detect numerous putative readthrough events (Figure 3). There are different mechanisms that can cause suppression and recording of stop codons that are detected as readthrough events at the genomic level. For example, in *Escherichia coli*, *Salmonella typhimurium*, and *Bacillus subtilis*, TGA is encountered less frequently than TAA and more frequently than TAG [38,39]. In *E. coli* and *S. typhimurium* TGA can be decoded at very low frequency; when this occurs, the amino acid inserted is tryptophan [40]. Thus, TGA is considered as a “leaky” termination codon [41]. In agreement with this, an unexpectedly high proportion of TGA nonsense mutations was obtained in a collection of chemically induced mutations in the *spoIIR* locus of *Bacillus subtilis* [42]. Six suppressors of the TGA mutations were isolated, and five of the suppressing mutations were mapped to the *prfB* gene encoding protein release factor 2. The five *prfB* mutations also resulted in suppression of the *catA86*-TGA mutation to 19–54% of the expression of *catA86*+, compared to the readthrough level of 6% in the *prfB*+ strain [42].

Other known mechanisms causing readthrough events at the genomic level are TAG to pyrrolysine translation via amber suppression [43], reassignment of TGA or TAG to selenocysteine [44], and TGA to selenocysteine and cysteine recoding [45]. It is not possible to estimate the overall impact of these or similar events [25]; however, it is likely that these events are functionally important and persist over long periods of evolutionary time. Nevertheless, results of the small-scale study of putative readthrough events suggest that many detected readthrough events exist for short periods of time; thus, they are likely to be deleterious at longer evolutionarily timescales. We assume that the vast majority of detected readthrough events do not have functional recording of in-frame stop codons.

Our results are consistent with previous studies of in-frame stop in eukaryotes. Some genes with in-frame stop codons in metazoan species exhibit evolutionary conservation of gene sequences, reduced nucleotide variability, excess synonymous over nonsynonymous nucleotide polymorphism, and other features that are expected in genes or DNA sequences that have functional roles [6]. The cytoplasmic inherited [PSI+] factor has long been known to reduce the efficiency of translation termination and, thereby, increase the readthrough of stop codons in the yeast *Saccharomyces cerevisiae* [46]. In addition, modest changes in the context surrounding the stop codon was shown to result in a substantial reduction in the efficiency of translation termination in eukaryotic organisms [47]. Thus, in-frame stop codons and associated readthrough events may represent a “short-term” mechanism of regulation, because lower levels of expression are expected, due to less efficient translation [48].

In general, gene dosage effect is likely to be an important factor in the evolution of gene families [49,50]. It was suggested that gene duplications that persist in an evolving lineage are beneficial from the time of their origin, due primarily to a protein dosage effect in response to variable environmental conditions [49]. However, a suppression of protein expression may be as important as an increase in protein expression. Thus, it is likely that a decrease in expression due to presence of in-frame stop codons is beneficial for functioning of prokaryotic cells (at least, for short periods of time). Importance of the gene downregulation in response to various factors has been shown for various pro- and eukaryotic species [51,52,53,54,55].

Future analyses of in-frame stop codons and readthrough events in pro- and eukaryotes can produce somewhat unexpected results. For example, recent studies suggested that efficiency of protein translation is likely to be associated with autism spectrum disorders (ASD) [56,57]. This observation connects environmental factors and genetic factors (SNPs and de novo mutations) because each can alter translation efficiency. Many SNPs and de novo mutations are positioned within the coding region of a gene, resulting in premature stop codons [58]. Thus, efficiency of readthrough events could have a functional effect on the protein translation and ASD phenotype [57].

Our results suggest frequent readthrough events in prokaryotes; however, many of them are likely to be deleterious at the scale of long-term evolution, and thus reversals (leading to full restoration of function) are advantageous, as suggested by the small-scale analyses (Figure 2 and Figure 6). It is hard to estimate the frequency of reversal (Figure 2), although it may be high, as suggested by the phyletic index (Figure 4). Taking into account that the fraction of independent events among all “orthologous” stop codons is small (0.015), many orthologous in-frame stop codons with phyletic index less than 1 may reflect the substantial impact of reversals.

In-frame stop codons are most frequently found in the COG functional category [X] “Mobilome: phages and transposons” (Supplementary Table S2). Prophage regions of prokaryotic genomes have at least two evolutionary fates: either domestication or pseudogenization [59,60]. The observed excess of orthologous stop codons in the COG category [X] (Supplementary Table S2) may be explained by the fact that many of them are pseudogenes [59] and do not experience reversals (Figure 2). For example, phages with numerous recorded stop codons (e.g., CrAssphages, [61]) may create an excess of in-frame stop. However, they are much more likely to become pseudogenes rather than “domesticated genes”, because of the presence of multiple in-frame stop codons in genes corresponding to recorded regions, e.g., “late” genes compared to phages with the standard genetic code. In general, the fraction of bacteria and phages with recorded stop codons is less than 1% (~0.044%) [19].

Frameshift mutations in protein-coding genes are caused by sequencing errors or programmed ribosomal frameshifting. Programmed ribosomal frameshift events are rare, but they are functionally important [62,63]. We avoided frameshift mutations by using the window (±30 nucleotides) surrounding in-frame stop codons. Even if frameshifts are present in our dataset, it will cause a false “positive selection” (dN/dS > 1), because the third position of affected codons (this position is known to be the most variable position) shifts to the first or second positions (these positions are known to be the most conserved positions) [30]. Thus, this can bias our estimates of dN/dS because dN values become artificially large and dS values become artificially small [64,65]. We observed the opposite trend (dN/dS < 1, Table 1 and Figure 5). Thus, frameshift mutations are not likely to affect our results and conclusions.

We removed *Mycoplasma* spp. genomes because this is the only known clade in the ATGC database [66] with the stop-codon recording. Our analysis of other potential stop-codon events did not reveal any deviations, except for expected *Mycoplasma* spp. (that we removed from our analyses) and a few *Rickettsia* spp. (Supplementary Figure S7). *Rickettsia* species have been known to contain numerous pseudogenes and are even used as model organisms for studies of pseudogene degradation [67]. Pseudogenes are expected to evolve according to the neutral mode of evolution (dN/dS ~ 1) [1]. Thus, pseudogenes cannot substantially bias our estimates of dN/dS in sequences surrounding in-frame stop codons (Table 1 and Figure 5).

There are at least four major sources of in-frame stop codons in our dataset: artifacts of annotations, sequencing errors, pseudogenes, and premature stop codons in functional genes (Supplementary Figure S2). All four types of sources cause problems for performing genome annotations and analyses. In this study, we used prokaryotic genomes with reliable annotations and a high-quality ATGC database, which is based on orthologous gene families [66]. All low-quality proteins were removed from this database. We also effectively removed effects of sequencing errors by using orthologous in-frame stop codons in two or more species (Supplementary Figure S1). These approaches are different from using (semi-)automated annotation pipelines, because all four sources of in-frame stop codons pose major challenges for these systems. Artifacts of previous annotations of closely related sequenced genomes may be, to some extent, resolved by using comparative genomics employed by some (semi-)automated annotation pipelines [68]. However, pseudogenes are an important source of in-frame stop codons in one or several species [67]. In addition to this problem, our analyses suggest that the readthrough mechanism is likely to function in many prokaryotes; thus, many putative “pseudogenes” with in-frame stop codons are functional genes instead. This poses a challenge for genome annotation pipelines, because in-frame stop codons without other signs of gene “degradation” (for example, multiple frameshifts or long deletions) appear to be poor markers of pseudogenization.

## 4. Materials and Methods

### 4.1. Identification of Nonsense Substitutions in Protein-Coding Genes

All *Mycoplasma* spp. genomes were removed from our analyses. All protein sequences from the ATGC database [66] were used as a query in TBLASTN (default parameters) searches against all ATGC genomes translated in six frames. When a stop codon was found aligned to an amino acid in the query and an accurate alignment was found 10 amino acids, upstream and downstream, the case was further considered. To reduce the possibility that a stop codon was the result of a sequencing error (Supplementary Figure S2), only orthologous cases with a stop codon in the same position in two or more independent genomes were considered. An example of orthologous an in-frame stop codon is shown in the Figure 2.

### 4.2. Phylogenetic Analysis and dN/dS Calculations

The maximum-likelihood phylogenetic tree was inferred, using the Tamura–Nei model in MEGA X [69]. Initial tree(s) for the heuristic search were obtained automatically by applying Neighbor-Join and BioNJ algorithms to a matrix of pairwise distances estimated by using the Maximum Composite Likelihood (MCL) approach, and then selecting the topology with superior log likelihood value. The analysis involved 18 nucleotide sequences. Codon positions included were 1st+2nd+3rd+Noncoding. All positions with less than 75% site coverage were eliminated, resulting in a total of 57 positions in the final dataset. CodeML [70] was used to estimate the dN/dS in a 30 bases window, upstream and downstream from the stop. Protein-coding genes are expected to be under purifying selection (dN/dS < 1). If sequences with stop codons are pseudogenes or represent errors of annotation, no purifying selection is expected. Significance of deviations of dN/dS values from 1 was estimated by using the two-tail *t*-test.

### 4.3. Double Substitution with Stop Intermediate Classification

A double substitution with a stop intermediate is a codon substitution where one of the single substitutions of that double would result in a stop codon. We subdivide double substitutions with stop intermediates into 3 subclasses: (a) S-stop-N—where one intermediate is the stop while the other intermediate is synonymous (S), and the final codon is nonsynonymous (N) to the original codon; (b) N-stop-Nn—where one intermediate is the stop, while the other is N, and also the final codon is N to the original, and the also the final codon is nonsynonymous (n) to the intermediate sense codon; (c) N-stop-Ns—where one intermediate is the stop, while the other is N, and also the final codon is N to the original, and the also the final codon is synonymous (s) to the intermediate sense codon. A subclass 3 can be viewed as a subset of subclass 2, where no selection is expected to affect the second step between the intermediate sense codon and the final codon (because they are synonymous and, thus, identical to the original codon. Thus, the distribution of DFs in subclass 3 is expected to be no different than that double synonymous substitutions, if nonsense mutations are so deleterious that they are completely purged by purifying selection.

### 4.4. Estimation of Selection on Double Substitutions with Stop Intermediates

Frequencies of double and single substitutions were calculated from the changes between triplets of closely related genomes, as previously described [33]. The DF, calculated as the ratio between the double frequency and the single plus double frequencies, was used as an estimate of the selection pressure on the second step of the double substitutions. When the DF was not different than that of the double synonymous controls, no selection was inferred; when the DF was higher than that of the double synonymous controls, then positive selection was inferred; and when the DF was lower than that of the double synonymous controls, the negative/purifying selection was inferred [33]. Both the *t*-test and signed rank test (two-tail tests) were used to assess the differences of DF between each class and the controls.

### 4.5. Analysis of Stop Codons within Protein-Coding Genes

To assess a distinction between real pseudogenes (genes with no function at the protein level) and apparent pseudogenes (genes possessing in-frame stop codons that, at least to some degree, can be translated or skipped during translation, resulting in a functional protein), we calculated the dN/dS measure of selection.

## Figures and Tables

**Table 1:** The dN and dS values for regions surrounding in-frame stop codons.

	dN/dS	dN	dS	*p* Value
TAA	0.7444	0.0023	0.0031	<0.001
TAG	0.8153	0.0023	0.0029	<0.001
TGA	0.6602	0.0028	0.0043	<0.001

## Supplementary Materials

The following are available online, at https://www.mdpi.com/1422-0067/22/4/1876/s1. Figure S1: Pipeline steps and the number of detected stop codons. Figure S2: Major technical problems. Figure S3: PSI-BLAST (iteration#2, default parameters) output for putative 3′-phosphoadenosine 5′-phosphosulfate sulfotransferase proteins. Figure S4: Distribution of nonsense substitution on species tree: phyletic index. Figure S5: Conceptual scheme of double substitution analysis and the double fraction (DF) measure. Figure S6: Double synonymous substitutions in adjacent codons used as null models and calculation of DF. Figure S7: Fraction of nonsense substitution compared to the genome size. Table S1: List of *Paenibacillus* species that are shown in Figure 1 and Figure 2. Table S2: Description of ATGC-COG functional categories where in-frame stop codons have been detected. Table S3: Statistics of double mutations and modes of selection.
