# Supplementary material for: Analysis of Stop Codons within Prokaryotic Protein-Coding Genes Suggests Frequent Readthrough Events
Source: Int J Mol Sci. 2021 Feb 14;22(4):1876. doi: 10.3390/ijms22041876 (PMC7918605; doi:10.3390/ijms22041876)
Supplement: Supplementary file 1 [file ijms-22-01876-s001.pdf]

Supplementary Materials

Analysis of stop codons within prokaryotic protein-coding genes suggests frequent readthrough events

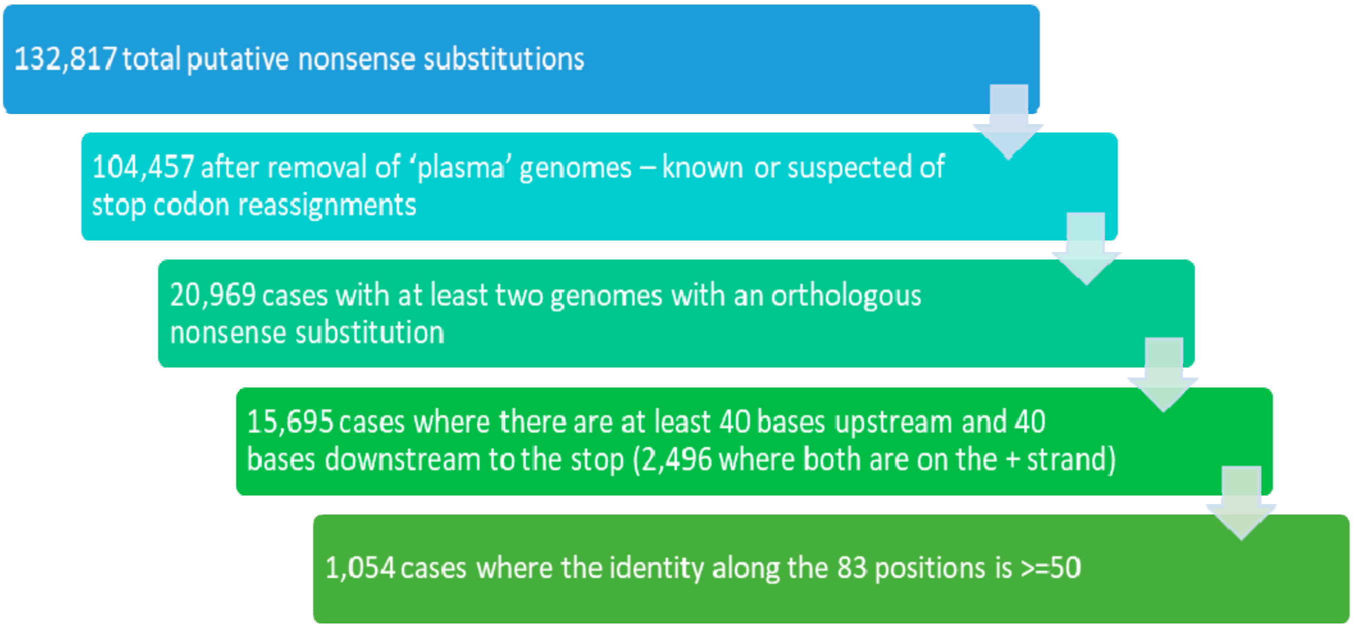

Figure S1. Pipeline steps and the number of detected stop codons.

Figure S2. Major technical problems. Blue indicates real genes, yellow indicates errors of prediction.

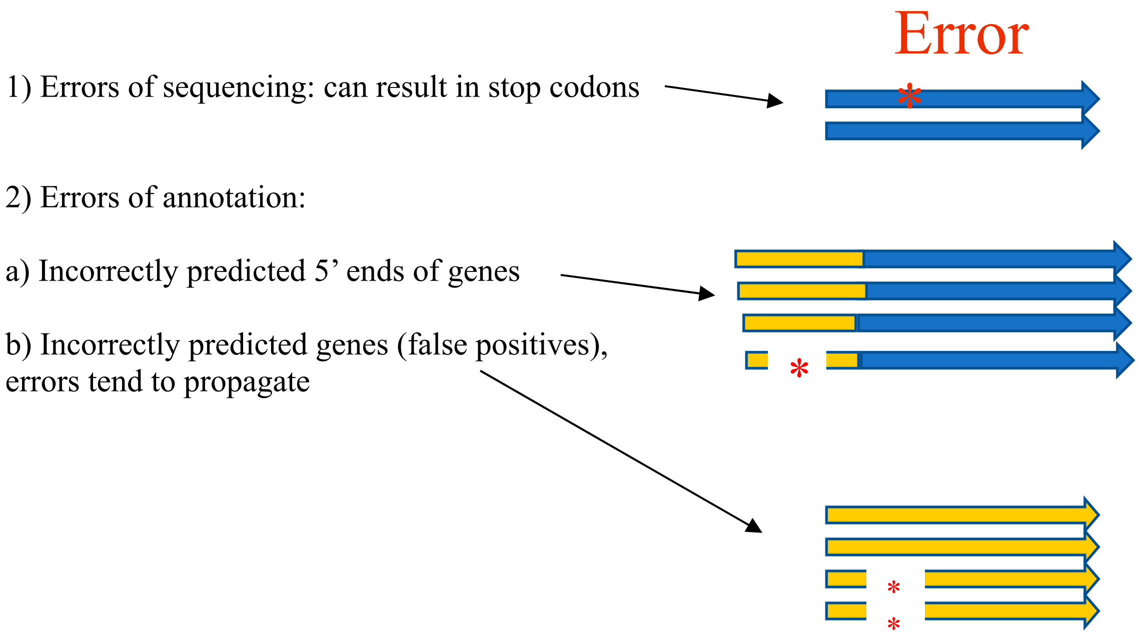

```
3'-phosphoadenosine 5'-phosphosulfate sulfotransferase [Paenibacillus thiaminolyticus]
Sequence ID: WP_174818191.1Length: 281 Number of Matches: 1
Alignment statistics for match #1
Score      Expect     Method                        Identities Positives  Gaps
204 bits(520)   2e-61     Composition-based stats.   58/251(23%)     101/251(40%)    12/251(4%)

Query  2      RQAFKQKLIEIIPALQGRVYDVQPPSQTAEEPYAVMALGEEIWKSSWAGYRQVVRIKLYA  61
              R   +Q+L++ IPA+ G++ D         +PY V+ +G E  ++ WAG    + +  YA
Sbjct  11     RSVIRQRLLDRIPAVNGKILDAGTAEAGEAKPYLVLTIGSETVENDWAGSSCKIEVAPYA  70

Query  62     GQAGLAQADVWANALISGLHREPVTGAGEDTSAFTAHYLGVRDAEKLDTVTGKAYRTLRF  121
                A L   D  A A+IS L R+ +T A    S     Y+G   ++ +D       R+++F
Sbjct  71     PPAELPHVDSLAAAVISALDRQRLTDAISGKSILF-RYIGT-GSDTVDEKLKAVARSVQF  128

Query  122    GVYVPETEGDSAVPANGATQPEEWLAALVRWTQKQLGETWSIYANAWPAQPGRHAVLWRM  181
               V+            +      + +AA+  WT+ +  E  +    AW       A+ WR+
Sbjct  129    EVFSL-------GWMSHTPLDPDPVAAMAAWTRARFPEIET-DPVAWNPSEDTPALYWRV  180

Query  182    SGCETRMAGASMYELRKRFIGHITAPDTTEENRAASALIEGFAAQIQLPLDQDKGRYMST  241
              +   +    +    +     GH+  P     +R     +   A   Q+ +       M
Sbjct  181    AAIRSIQTVSQGAWIEATLRGHMLVPHAEARSRWLDLTVRQLALDGQVRM--LDQSPMLI  238

Query  242    AEASADLQADA  252
                 SAD   DA
Sbjct  239    QSVSADGTQDA  249
```

Figure S3. PSI-BLAST (iteration#2, default parameters) output for putative 3'-phosphoadenosine 5'-phosphosulfate sulfotransferase proteins (Query: WP_058711956 and Sbjct: WP_036625970).

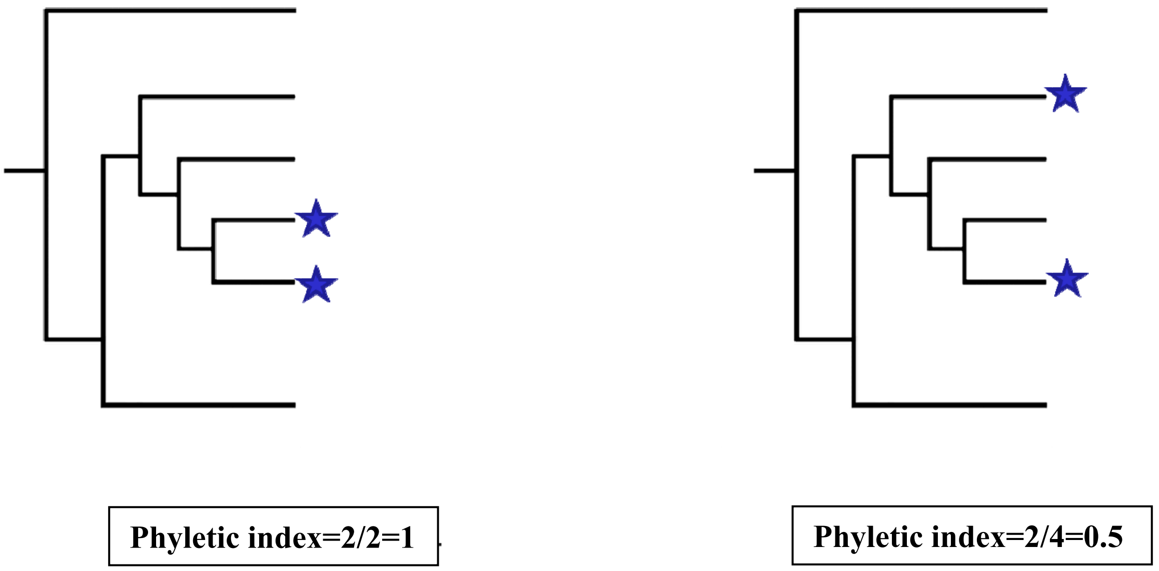

Figure S4. Distribution of nonsense substitution on species tree: phyletic index.

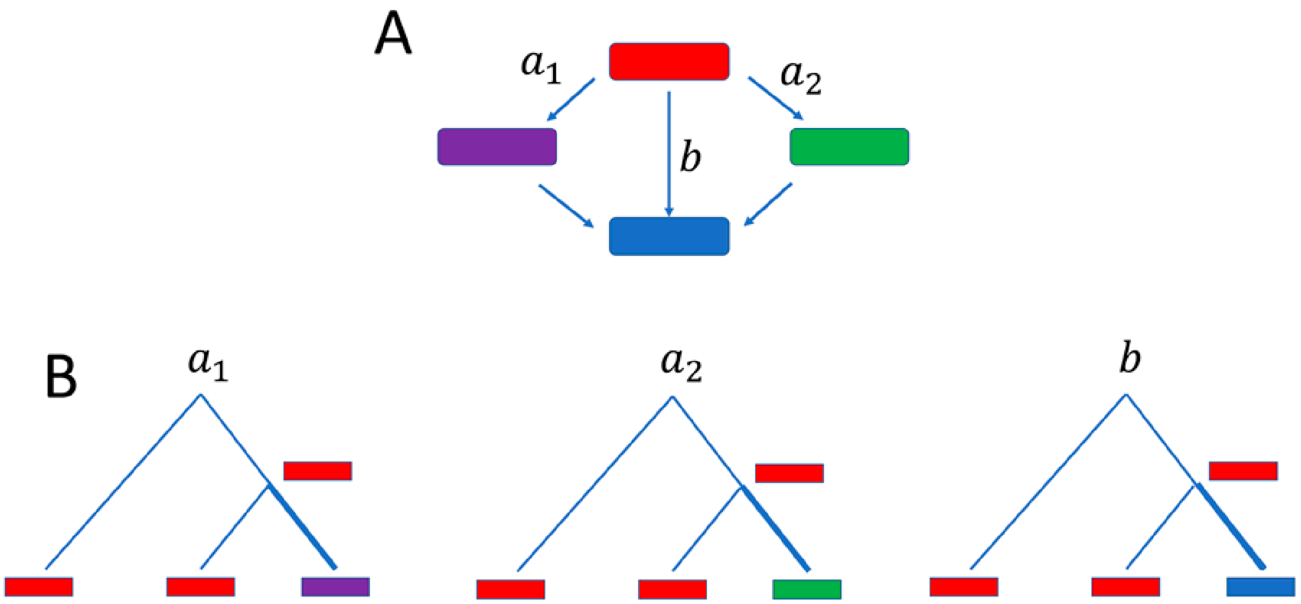

$$DF = \frac{b}{a_1 + a_2 + b}$$

Figure S5. Conceptual scheme of double substitution analysis and the **double fraction (DF) measure.** (**A**) Point mutations are assumed to appear one at a time, such that observed double substitutions (b) occur through intermediate single substitution states. For each double substitution instance, there are two possible single substitution trajectories (a1 and a2). (**B**) Instances of single or double substitutions are inferred from the genomic data by construction of genomes triplets and relying on parsimony principle (see the "Methods" section). In brief, the parsimony principle implies that mutations occur along the thick branches in the trees shown in (**B**). The double fraction is defined as the ratio between the number of double substitution instances b and the sum of relevant single (a1 + a2) and double (b) substitution instances.

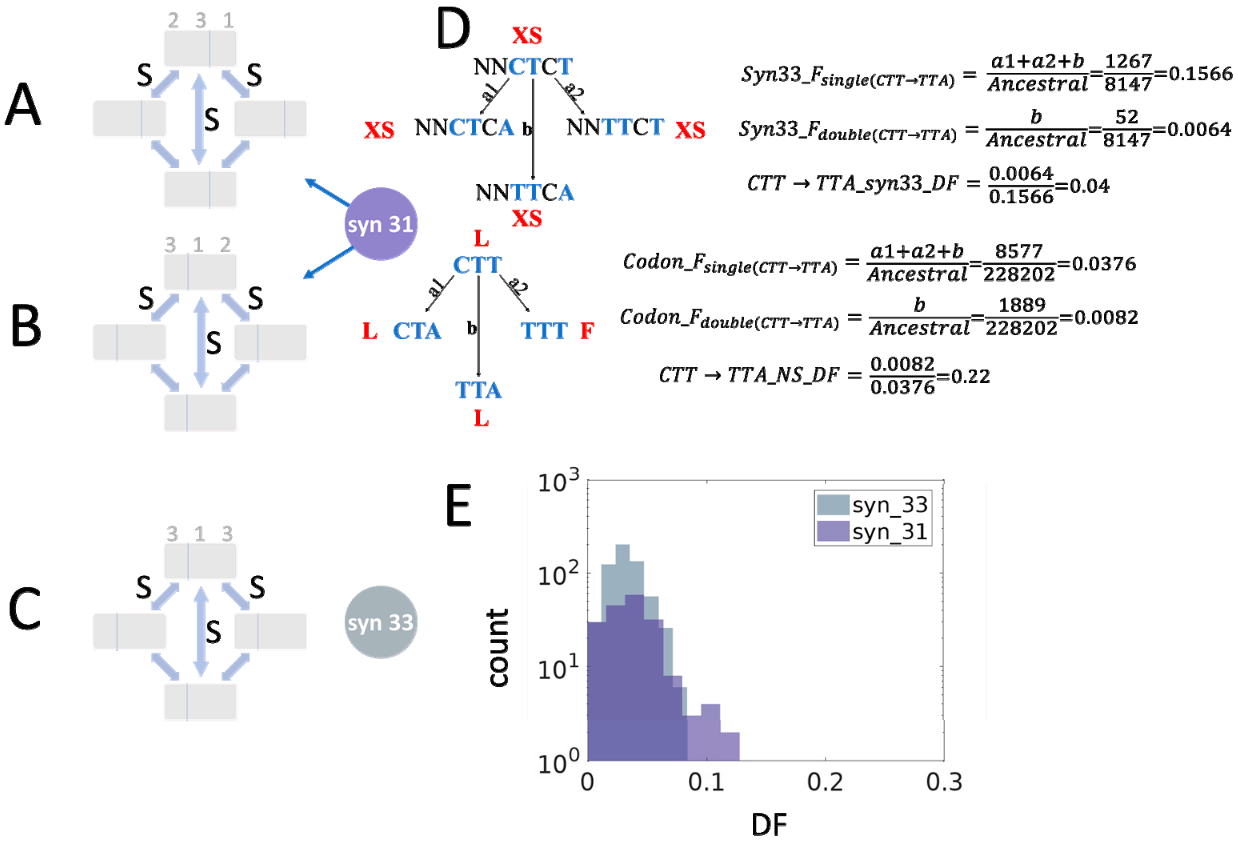

Figure S6. Double synonymous substitutions in adjacent codons used as null models and calculation of DF. (**A**) A constant 2nd codon positions followed by a 4-fold degenerate site in the 3rd codon positions which is followed by a 2-fold degenerate site in the 1st codon position of the next codon (**B**) A 4-fold degenerate site in the 3rd codon position followed by a 2-fold degenerate site in the 1st codon position of the next codon, which is followed by a constant base in the 2nd codon position of the second codon. (**C**) A 4-fold degenerate site in the 3rd codon position followed by a constant 1st codon position in the second codon of which the 2nd position is disregarded and by a 4-fold degenerate site in the 3rd codon position. (**D**) An example calculation of the DF under the null model syn_33 and an example calculation of the DF in an NS codon double substitution. (**E**) Comparison of DF between the two null models, syn_31 (adjacent synonymous substitutions) and syn_33 (non-adjacent synonymous substitutions).

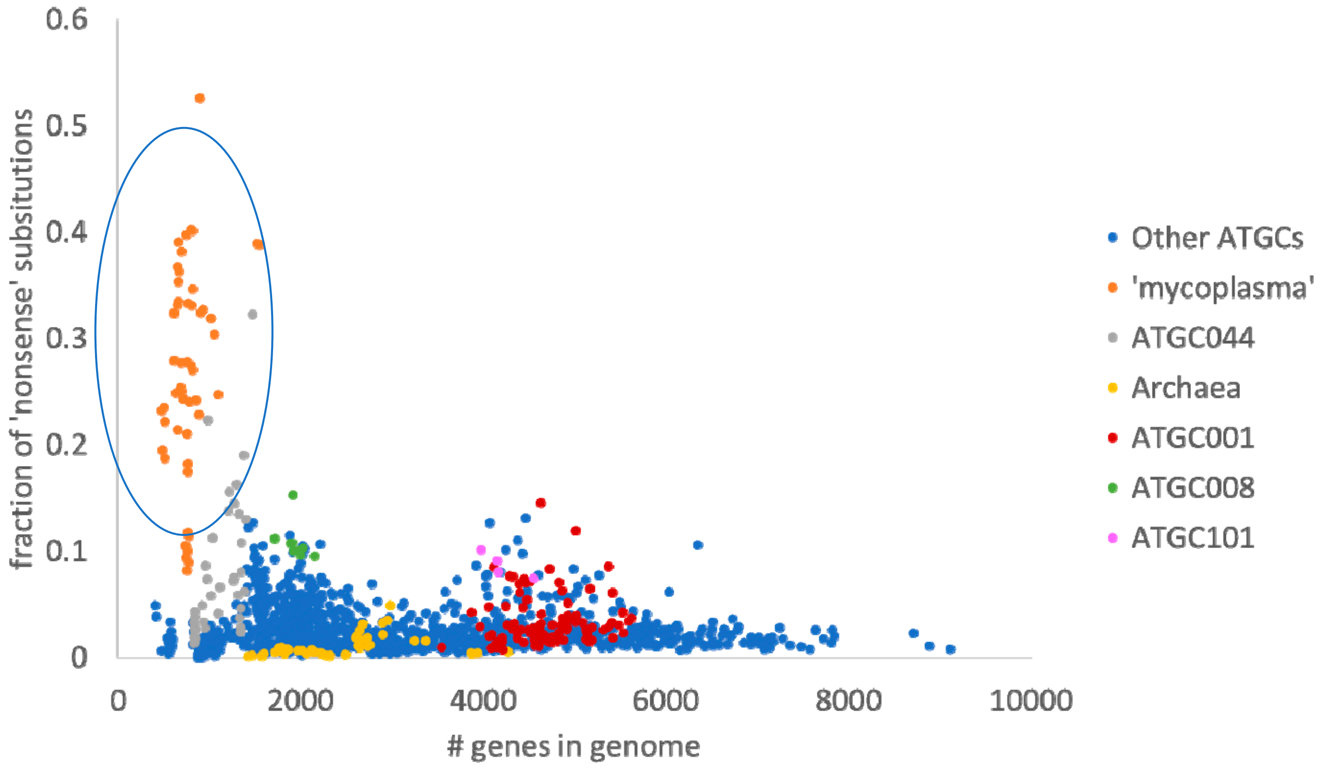

0.6
0.5
0.4
0.3
0.2
0.1
0
fraction of 'nonsense' subsitutions
0
2000
4000
6000
8000
10000
genes in genome
Other ATGCs
'mycoplasma'
ATGC044
Archaea
ATGC001
ATGC008
ATGC101

Figure S7. Fraction of nonsense substitution compared to the genome size. ATGC001 is the *Enterobacter* spp. group; ATGC008 is the *Streptococcus* spp. group; ATGC044 is the *Rickettsia* spp. group; ATGC101 is the *Dickeya* spp. group. The removed 'mycoplasma' group corresponds to ATGC32-ATGC40. "Other ATGCs" corresponds to all other ATGCs from the ATGC database used in this study. The blue ellipse shows obvious outliers.

Table S1. List of *Paenibacillus* species that are shown in Figures 1 and 2.

CP006941	*Paenibacillus polymyxa*
CP017967	*Paenibacillus polymyxa*
CP011512	*Paenibacillus peoriae*
CP017968	*Paenibacillus polymyxa*
CP000154	*Paenibacillus polymyxa*
CP011420	*Paenibacillus polymyxa*
CP015423	*Paenibacillus polymyxa*
CP034141	*Unidentified Paenibacillus sp.*
HE577054	*Paenibacillus polymyxa*
CP042272	*Paenibacillus polymyxa*
CP040829	*Paenibacillus polymyxa*
CP025957	*Paenibacillus polymyxa*
CP009909	*Paenibacillus polymyxa*
CP006872	*Paenibacillus polymyxa*
CP013203	*Unidentified Paenibacillus sp.*
CP003107	*Paenibacillus terrae*
CP020028	*Paenibacillus kribbensis*
CP045298	*Paenibacillus brasilensis*

Table S2. Description of ATGC-COG functional categories where in-frame stop codons have been detected.

COG functional category	Number of orthologous genes containing in-frame stop codons	Number of singletons
[X] Mobilome: phages and transposons	1311	8578
[R] General function prediction only	610	5516
[G] Carbohydrate transport and metabolism	508	6689
[E] Amino acid transport and metabolism	472	7600
[S] Function unknown	348	2003
[T] Signal transduction mechanisms	371	5389
[K] Transcription	363	2836
[M] Cell wall/membrane/envelope biogenesis	335	3218
[L] Replication, recombination, and repair	306	3269
[P] Inorganic ion transport and metabolism	287	4027

Orthologous genes containing in-frame stop codons – in-frame stop codons that are present within orthologous protein coding genes shared by two or more bacterial species. Singletons – in-frame stop codons that were found only once (do not have orthologs). The most frequent categories are shown. ATGC-COG functional categories are ordered according to the "Number of orthologous genes containing in-frame stop codons" column.

Table S3. Statistics of double mutations and modes of selection

**Codon change**		**Null model - double synonymous changes**			Number of observed changes in codons			Fisher test	Class	mode of selection
**ancestral**	**derived**	**double**	**single**	ancestral	double	single	ancestral			after Bonferoni
CAG	TCG	39	1023	14713	201	1288	630066	1.40E-18	XNnN	+
TGC	TCA	56	2067	184686	36	176	154212	1.02E-15	XNsN	+
TAT	GAA	23	759	13413	52	243	325159	4.22E-15	XNnN	+
TAC	GAA	22	711	13176	28	126	336894	1.71E-10	XNnN	+
TAC	AAA	14	524	13176	30	144	336894	2.62E-10	XNnN	+
TAT	AAA	47	828	13413	63	323	325159	1.75E-09	XNnN	+
TGG	GGA	329	7154	51165	38	252	350601	7.19E-09	XNsN	+
CAG	TAT	54	3107	72658	68	1514	630066	2.83E-07	XNnN	+
TCG	CAG	20	542	7574	160	1414	314455	3.22E-07	XNnN	+
TGC	TTA	9	1223	184686	9	150	154212	5.24E-05	XNnN	+
AAG	TCG	18	703	20988	91	1354	534568	9.50E-05	XNnN	+
TAT	GAG	17	641	13413	20	211	325159	1.99E-04	XNnN	+
TAT	AAG	22	691	13413	28	288	325159	2.10E-04	XNnN	+
TAC	AAG	16	477	13176	17	131	336894	3.73E-04	XNnN	+
TGG	AGA	242	6275	51165	12	95	350601	7.54E-04	XNsN	+
TAC	GAG	29	669	13176	15	113	336894	1.82E-03	XNnN	=
CAG	TAC	163	7212	72658	62	1844	630066	1.19E-02	XNnN	=
TAC	CAG	62	2089	13176	52	1113	336894	2.13E-02	XNnN	=
CAA	TAT	51	990	7534	70	879	270512	2.40E-02	XNnN	=
GAG	TCG	6	324	8129	129	3634	668983	1.46E-01	XNnN	=
TGC	GGA	120	4369	84580	11	263	154212	1.82E-01	XNnN	=
TAC	CAA	54	2130	13176	34	1095	336894	3.64E-01	XNnN	=
TGT	CGA	270	9300	100899	4	98	60699	5.38E-01	XNnN	=
AGA	TGC	100	3299	54066	5	161	94676	8.16E-01	XNnN	=
TGG	CGA	795	11823	51165	27	399	350601	9.19E-01	XNnN	=

TAC	TTA	2	134	8037	29	1566	336894	1.00E+00	XNnN	=
TTA	TAC	5	129	3439	25	610	269701	1.00E+00	XNnN	=
GGA	TGG	267	8630	56720	31	23114	186121	9.69E-113	XSN	-
AGA	TGG	252	5843	54066	7	7916	94676	1.08E-81	XSN	-
CGA	TGG	752	11369	53638	9	3856	38022	2.66E-78	XSN	-
GGA	TGC	131	3465	56720	5	8789	186121	5.25E-64	XSN	-
GGA	TGT	100	2699	56720	15	8775	186121	2.28E-46	XSN	-
CGA	TGC	302	7107	53638	8	2152	38022	2.08E-24	XSN	-
CGA	TGT	277	5907	53638	8	1619	38022	2.22E-19	XSN	-
TCA	TGC	16	212	4610	26	3930	122445	1.69E-10	XSN	-
GAA	TAT	24	548	11884	71	4927	747383	2.03E-05	XNnN	-
GAA	TAC	15	547	11884	42	5754	747383	8.62E-05	XNnN	-
CAA	TAC	63	1024	7534	36	1147	270512	1.39E-03	XNnN	=
AGA	TGT	241	3795	54066	2	154	94676	8.10E-03	XNnN	=
GAG	TAT	15	814	70692	37	4449	668983	1.23E-02	XNnN	=
TCA	TAC	3	179	4610	13	3917	122445	3.12E-02	XSN	=
AAA	TAC	30	983	20660	36	1819	574412	9.04E-02	XNnN	=
AAG	TAT	18	462	12030	28	1157	534568	1.37E-01	XNnN	=
GAG	TAC	29	3740	70692	30	5831	668983	1.40E-01	XNnN	=
AAA	TAT	58	1291	20660	79	2293	574412	1.47E-01	XNnN	=
TAT	CAA	59	1249	13413	63	1745	325159	1.60E-01	XNnN	=
AAG	TAC	18	558	12030	18	886	534568	1.71E-01	XNnN	=
TCG	GAG	11	271	7574	105	3891	314455	1.86E-01	XNnN	=
TCG	AAG	12	191	7574	65	1471	314455	2.75E-01	XNnN	=
TGC	AGA	108	3164	84580	8	355	154212	3.43E-01	XNnN	=
TAT	CAG	36	1114	13413	47	1729	325159	4.94E-01	XNnN	=
TGC	CGA	315	13019	84580	6	286	154212	1.00E+00	XNsN	=
TGT	GGA	179	5403	100899	3	117	60699	1.00E+00	XNsN	=
TTA	TGC	3	106	3439	16	601	269701	1.00E+00	XNnN	=

+, positive; −, negative; =, neutral selection cannot be rejected.
